# Supplementary material for: A User-Centric 3D-Printed Modular Peristaltic Pump for Microfluidic Perfusion Applications
Source: Micromachines (Basel). 2023 Apr 25;14(5):930. doi: 10.3390/mi14050930 (PMC10222968; doi:10.3390/mi14050930)
Supplement: Supplementary file 1 [file micromachines-14-00930-s001.zip › micromachines-2316394-supplementary.pdf]

Supplementary Information

A User-Centric 3D Printed Modular Peristaltic Pump For Microfluidic Perfusion Applications

Jorge A. Catano1,2, Steven Farthing1, Zeus Mascarenhas 1, Nathaniel Lake1, Prasad KDV Yarlagadda1,2, Zhiyong Li1,2, Yi-Chin Toh1-4*

Supplementary Tables

SI Table 1. Electronic controller module part list with a description of each part needed for the manufacturing of the controller, part numbers, approximate USD cost, quantity needed, supplier and direct website link.

Parts	Part Number	Cost Per Unit	QTY Per 1	Supplier	Links
Jiffy box	HB6013	$4.50	1	Jaycar	https://www.jaycar.com.au/jiffy-box-black-130-x-68-x-44mm/p/HB6013?pos=7&queryId=1b8fd4dd0d182d71922f0e1232effa72&sort=relevance
Arduino	Arduino Leonardo	$29.95	1	Jaycar	https://www.jaycar.com.au/duinotech-leonardo-r3-development-board-for-arduino/p/XC4430?pos=2&queryId=31d6b1ff9d4951e08c78a6060392ea93
4 Pin Microphone Panel Male Connector	PP2010	$3.50	2	Jaycar	https://www.jaycar.com.au/4-pin-microphone-panel-male-connector/p/PP2010?
4 Pin Microphone Line Female Connector	PS2012	$3.75	2	Jaycar	https://www.jaycar.com.au/4-pin-microphone-line-female-connector/p/PS2012?pos=17&queryId=423d71ae7f3ec5fc7ef98cc57fd58aaf&sort=relevance
4 Core Alarm Cable	WB1590	$0.95	2	Jaycar	https://www.jaycar.com.au/4-core-alarm-cable-sold-per-metre/p/WB1590?pos=2&queryId=bbc8bfc04667b0ec7be938af4cd48c8d
Digital Rotation Sensor for Arduino	XC3736	$5.95	1	Jaycar	https://www.jaycar.com.au/digital-rotation-sensor-for-arduino/p/XC3736?gclid=CjwKCAiA4rGCBhAQEiwAelVti5k8_lwFW9G4vv1sIW-exjxakTIlidZePvHi8l7qwy2zoponJiqhUxoCrQgQAvD_BwE
Red Miniature Pushbutton	SP0710	$1.95	1	Jaycar	https://www.jaycar.com.au/red-miniature-pushbutton-spst-momentary-action-125v-1a-rating/p/SP0710?pos=20&queryId=98c5a86ad1f15102a2c990ac269b0efc&sort=relevance
S/TAP Screw #4x9MM 25Pk	HP0565	$4.95	0.16	Jaycar	https://www.jaycar.com.au/no-4-x-9mm-steel-self-tapping-screws-pk-25/p/HP0565?pos=1&queryId=a0fd0b541bc3e858c0ca2067fa042cb0&sort=relevance
2.1mm DC Power Line Connector	PP0510	$2.25	1	Jaycar	https://www.jaycar.com.au/2-1mm-dc-power-line-connector/p/PP0510?pos=1&queryId=9c21b9ebb1c1d34dd37467aacca3a764&sort=relevance
2.1mm Bulkhead Male DC Power Connector	PS0522	$2.95	1	Jaycar	https://www.jaycar.com.au/2-1mm-bulkhead-male-dc-power-connector/p/PS0522?pos=1&queryId=af4eb33f421859e7a1f25b8111d136c0&sort=relevance
10 Pin 0.1 Header with Crimp Pins - 2.54 pitch	HM3410	$1.40	2	Jaycar	https://www.jaycar.com.au/10-pin-0-1-header-with-crimp-pins-2-54-pitch/p/HM3410?pos=13&queryId=86b6a81b4ce99cece7570fd286df61fe&sort=relevance
4 Pin 0.1 Straight Locking Header -	HM3414	$0.50	5	Jaycar	https://www.jaycar.com.au/4-pin-0-1-straight-locking-header-2-54-pitch-single/p/HM3414?

2.54 pitch - Single					
4 Pin 0.1 Header with Crimp Pins - 2.54 pitch	HM3404	$0.85	5	Jaycar	https://www.jaycar.com.au/4-pin-0-1-header-with-crimp-pins-2-54-pitch/p/HM3404?pos=16&queryId=86b6a81b4ce99cece7570fd286df61fe&sort=relevance
2 Way 5.08mm Weidmuller PCB Mount Screw Terminal	HM3130	$1.35	1	Jaycar	https://www.jaycar.com.au/2-way-5-08mm-weidmuller-pcb-mount-screw-terminal/p/HM3130?pos=15&queryId=c8cd2653ed6547265977a4f0cae79659&sort=relevance
Micro B Round Panel Mount Extension Cable - 30cm	ADA4217	$11.20	1	CORE Electronics	https://core-electronics.com.au/micro-b-round-panel-mount-extension-cable-30cm.html?utm_source=google_shopping&gclid=Cj0KCQjwse-DBhC7ARIsAI8YcWJI8VGWiRAv2th1gMikkUrO0ogzJtxUBTk-JdXXzqq57BG-D_v6qJYaAqMjEALw_wcB
USB DIY Connector - MicroB Female Plug	ADA1829	$2.13	1	CORE Electronics	https://core-electronics.com.au/usb-diy-connector-microb-female-plug.html
USB DIY Slim Connector Shell - MicroB Plug	ADA1826	$2.13	1	CORE Electronics	https://core-electronics.com.au/usb-diy-slim-connector-shell-microb-plug.html
Molex, KK 254 Female Connector Housing, 2.54mm Pitch, 5 Way, 1 Row	679-5385	$0.40	2	RS Components	https://au.rs-online.com/web/p/wire-housings-plugs/6795385/?bid=3390032&cid=rscDM251&cm_mmc=AU-EM-_-undefined_16%2Far%2FTue%20-_-rscDM251-_-ProductDescriptionLink
Molex, KK 254, 6410, 5 Way, 1 Row, Straight Pin Header	670-1336	$1.75	2	RS Components	https://au.rs-online.com/web/p/pcb-headers/6701336/?bid=3390032&cid=rscDM251&cm_mmc=AU-EM-_-undefined_16%2Far%2FTue%20-_-rscDM251-_-ProductDescriptionLink
Heat Shrink		$15.69	1	eBay	https://www.ebay.com.au/itm/530-Pcs-Heat-Shrink-Tubing-Tube-Assortment-Wire-Cable-Insulation-Sleeving-Kit-/184675826690?hash=item2aff897c02
9V 1A AC/DC Power Supply		$9.12	1	eBay	https://www.ebay.com.au/itm/AU-Power-Supply-AC-adapter-DC-3V-4-5V-5V-6V-7-5V-8V-9V-10V-12V-15V-1A-2A-1000mA/224041651889?hash=item3429ec4eb1:g:pj0AAOSwUvldBylt
Motor Driver	A3967 Easy Driver Stepper Motor Driver	$7.50	1	eBay	https://www.ebay.com.au/itm/A3967-Easy-Driver-Stepper-Motor-Driver-V4-4-Pin-Header-For-Arduino/273712799067?_trksid=p2485497.m4902.l9144
OLED Screen	OLED Display 0.96" 128x64 White I2C IIC SSD1306	$9.05	1	eBay	https://www.ebay.com.au/itm/OLED-Display-0-96-128x64-White-I2C-IIC-SSD1306-Arduino-Raspberry-Pi/273746793961?_trksid=p2485497.m4902.l9144
2 mm Thick Acrylic Sheet	210x148x2 mm Sheet clear acrylic	$11.80	1	eBay	https://www.ebay.com.au/itm/CLEAR-Acrylic-Perspex-Sheet-1-10mm-thick-Up-to-20-OFF-Factory-Price-FREE-POST/143739654414?ssPageName=STRK%3AMEBIDX%3AIT&_trksid=p2057872.m2749.l2649
Ribbon Cable	WM4502	$2.75	1m	Jaycar	https://www.jaycar.com.au/16-way-idc-ribbon-cable-sold-per-metre/p/WM4502
Hook-up Wire (Red)	WH3040	$0.65	1m	Jaycar	https://www.jaycar.com.au/red-heavy-duty-hook-up-wire-sold-per-metre/p/WH3040

Hook-up Wire (Black)	WH3041	$0.65	1m	Jaycar	https://www.jaycar.com.au/black-heavy-duty-hook-up-wire-sold-per-metre/p/WH3041
40-Pin Header Strip	HM3212	$1.10	1	Jaycar	https://www.jaycar.com.au/40-pin-header-terminal-strip/p/HM3212
RS PRO Hybrid, Permanent Magnet Stepper Motor, 3.8 V, 5mm Shaft Diameter	535-0344	$113.21	1	RS Components	https://au.rs-online.com/web/p/dc-motors/5350344/?fbclid=IwAR0klaVS2EncJHEWKZjJ-8Wv3oWsjMBB97F5F7wpd3UY79otfh7LUa3_avU

Table 2. Standard Parts list - Description of independent standard components used for assembly.

**Parts**	**Part Number**	**QTY Per 1**	**Supplier**
M3 hex screw 20mm	Na	5	Na
M2.5 hex screw 6mm	Na	4	Na
M2 hex crew s8mm	Na	3	Na
NSK Deep Groove Ball Bearing - Plain Race Type, 5mm I.D, 16mm O.D	625-2RS	1	NSK

Supplementary figures

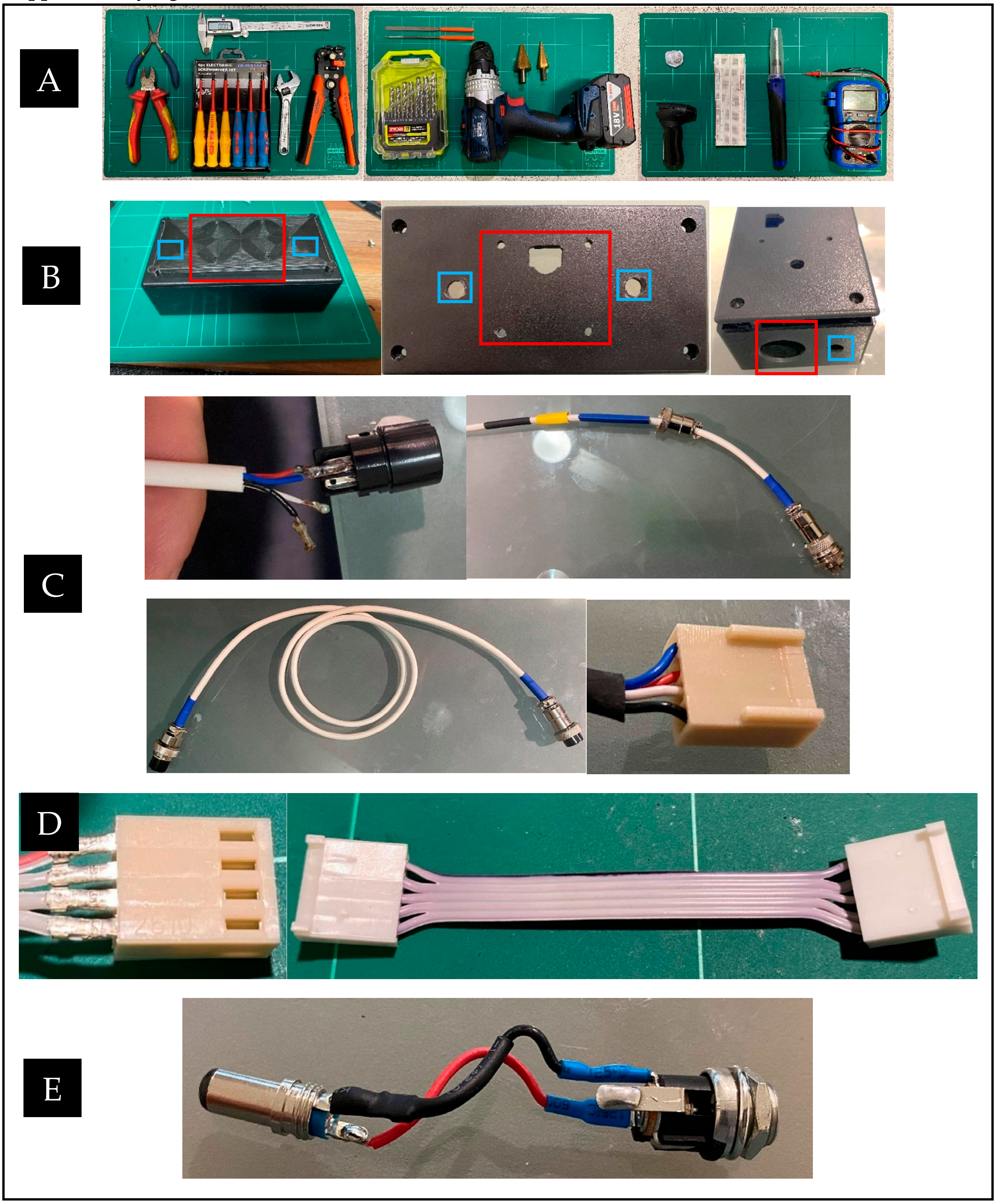

SI Figure 1. Recommended tools and parts used for prototyping and assembly of the electronic controller module. (A) Recommended tools for electronic controller module fabrication**. (B)** Plastic box cut-outs for external wire connections. **(C)** 4-pin panel wires assembly. **(D)** Ribbon cable assembly. **(E)** DC Socket extension assembly.

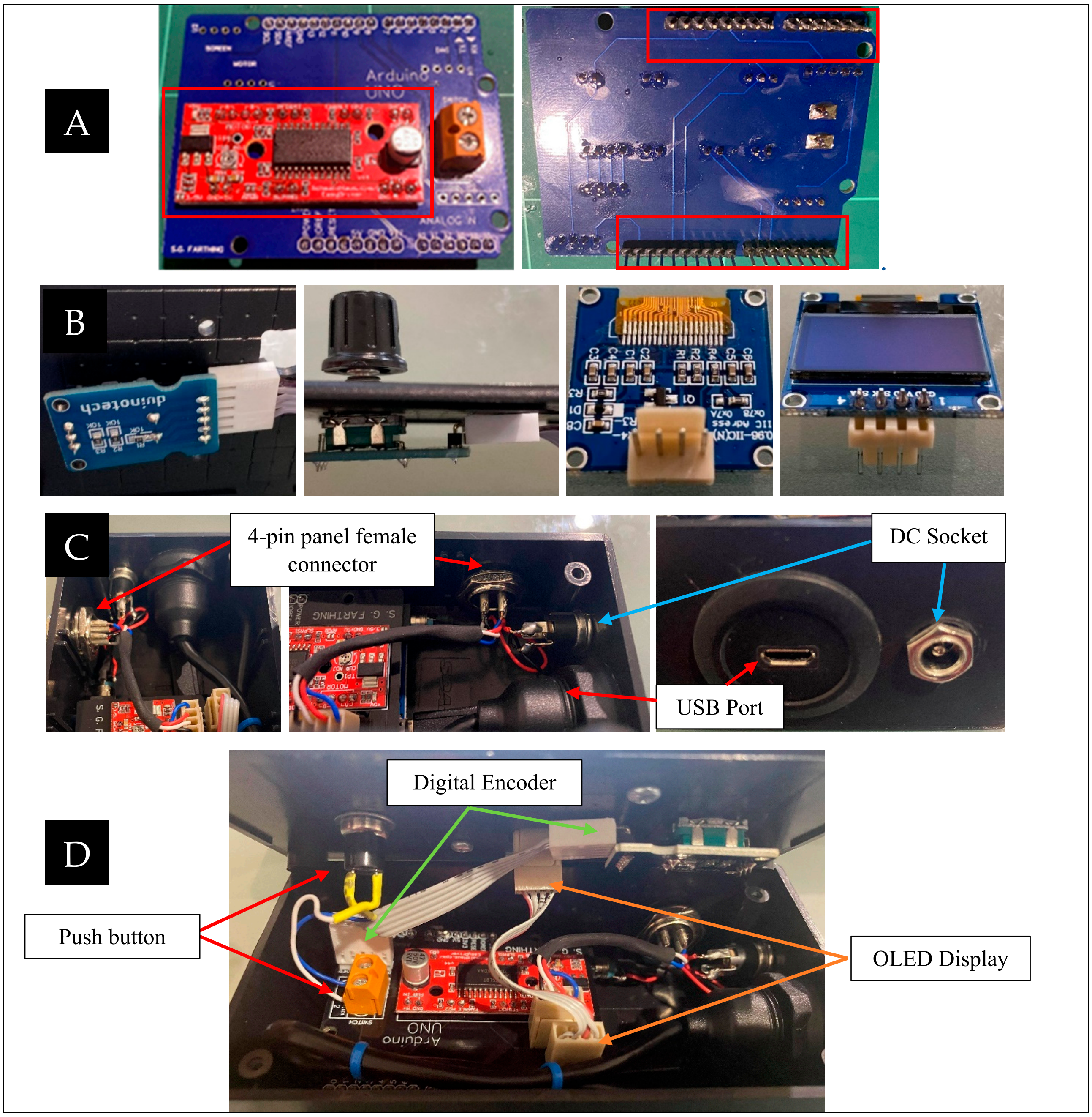

SI Figure 2. Electrical connections within the electronic control module. (A) Soldering of motor driver and header pins to custom PCB. **(B)** Mounting of Digital Encoder and OLED display**. (C)** The 4-pin female panel, USB port extension and DC socket extension assembly. **(D)** The connection assembly of the push button, digital encoder, and OLED display.

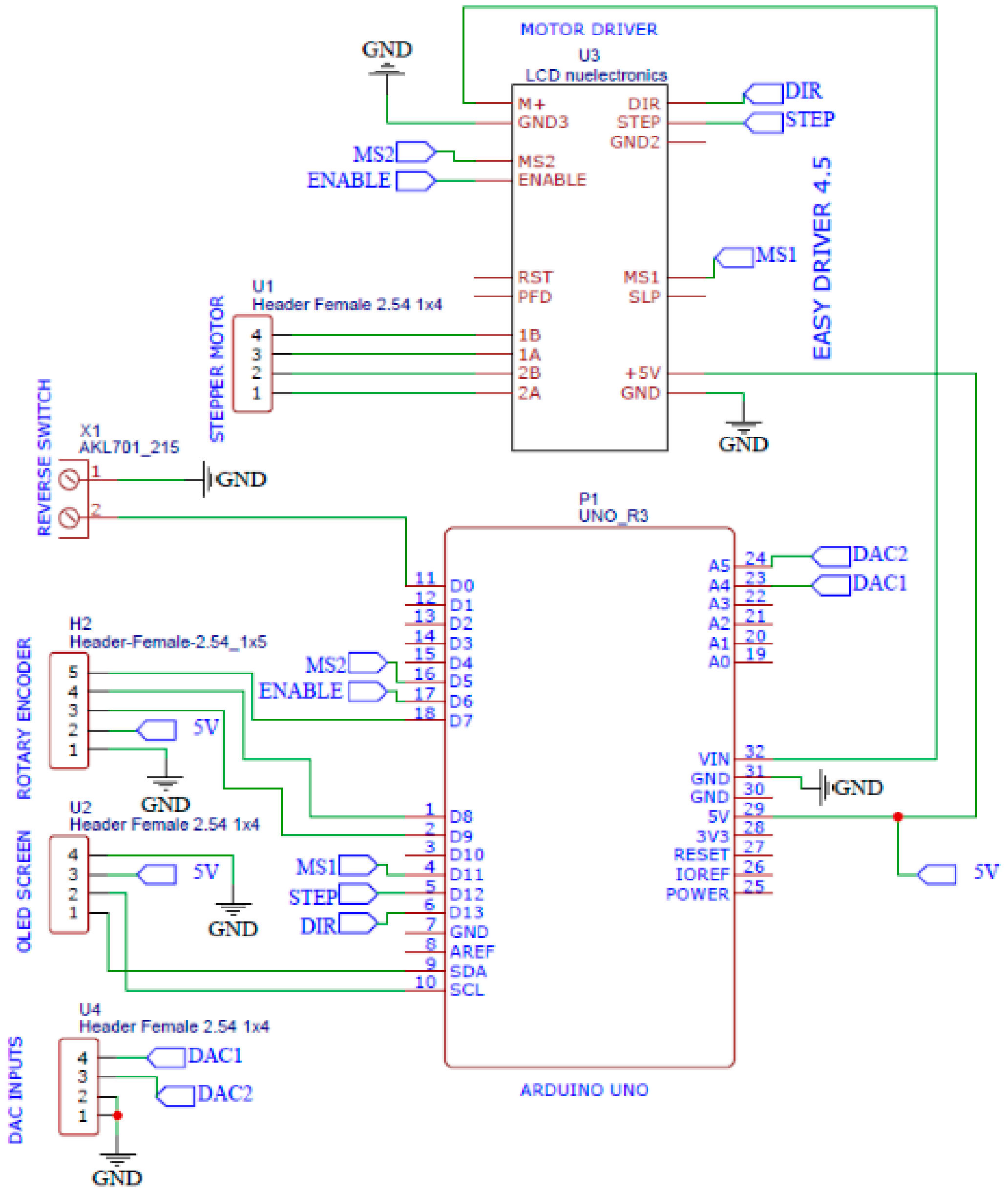

SI Figure 3. Diagram showing connections between different components in the electronic controller module. The designed electronic circuit schematic of routing and specific pins assigned for the different components connected to the Arduino microcontroller, DC barrel and motor driver. The push button, rotary encoder, and motor driver were connected to the digital pins of the Arduino microcontroller, while the OLED display screen used the serial clock and data pins. The rotary encoder and OLED display operated at 5V and used the 5V output pin from the Arduino microcontroller, while the motor driver operated at the 9V provided by the DC barrel power connector through the VIN pin.

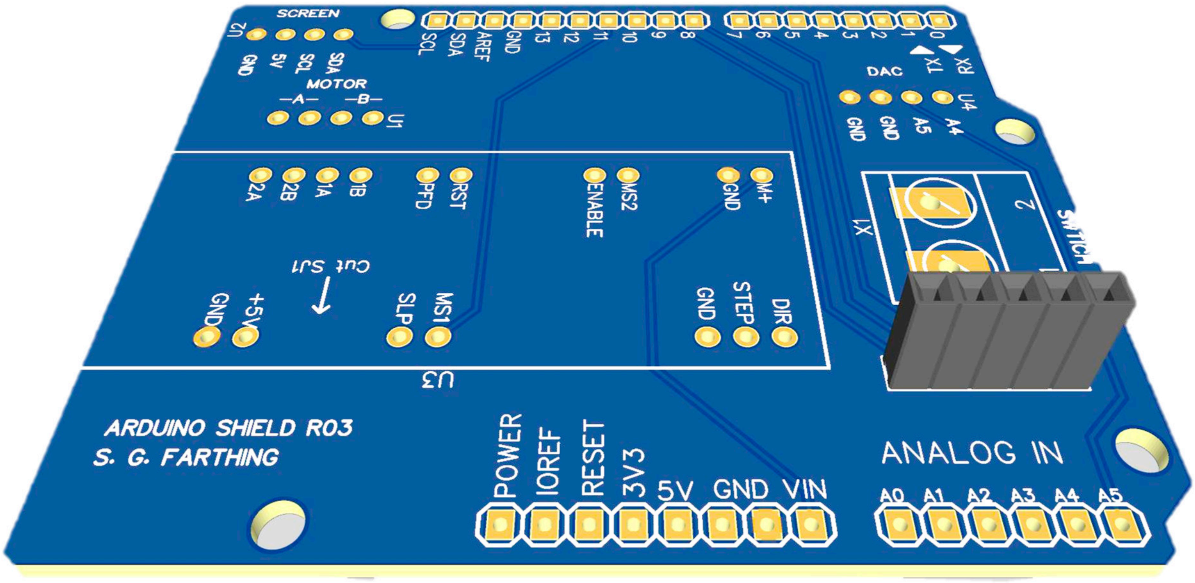

SI Figure 4. Custom PCB design. The PCB was fabricated as double-sided, meaning the top and bottom layers were used for routing. On the top layer, three header pins for ribbon cables were soldered to connect the OLED screen, rotary encoder, and motor cable connections. The motor driver was located on the top layer of the PCB and soldered in place. The external ports were wired and connected to the PCB board with plug-and-play connections for easy disconnection. The female panel connector located on the outer side of the housing was also wired to the PCB to connect the motor cable.

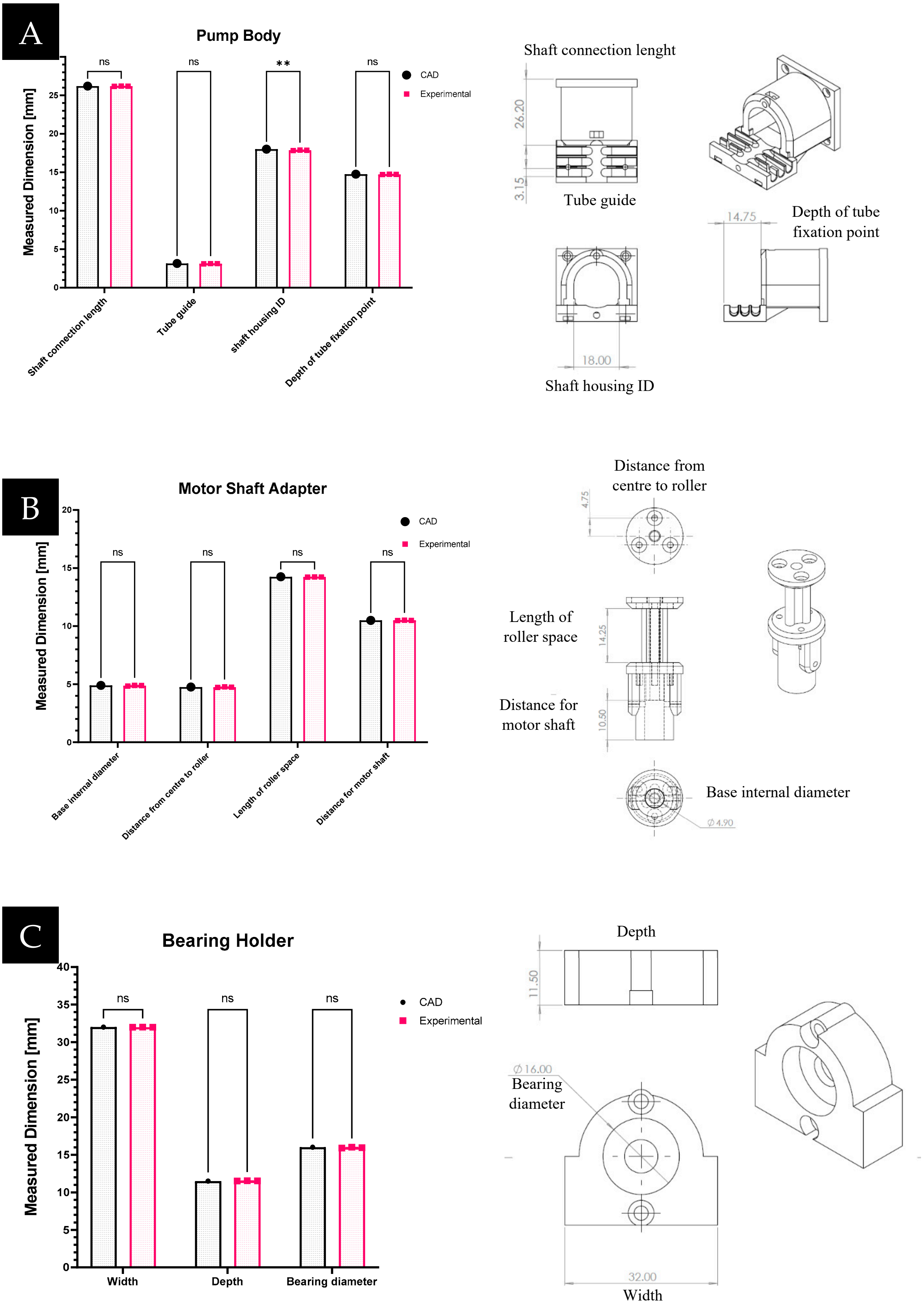
A
Pump Body
Measured Dimension [mm]
ns
ns
**
ns
CAD
Experimental
Shaft connection length
Tube guide
shaft housing ID
Depth of tube fixation point
Shaft connection lenght
26.20
3.15
Tube guide
14.75
Depth of tube fixation point
18.00
Shaft housing ID
B
Motor Shaft Adapter
Measured Dimension [mm]
ns
ns
ns
ns
CAD
Experimental
Base internal diameter
Distance from centre to roller
Length of roller space
Distance for motor shaft
Distance from centre to roller
4.75
Length of roller space
14.25
Distance for motor shaft
10.50
Base internal diameter
Ø 4.90
C
Bearing Holder
Measured Dimension [mm]
ns
ns
ns
CAD
Experimental
Width
Depth
Bearing diameter
Depth
11.50
Ø 16.00
Bearing diameter
32.00
Width

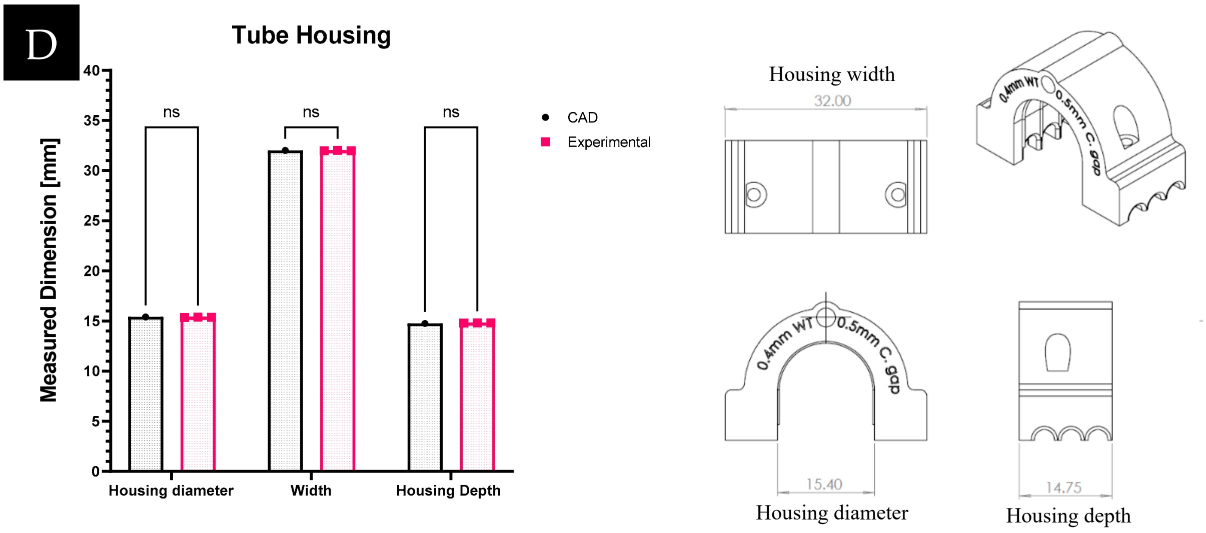

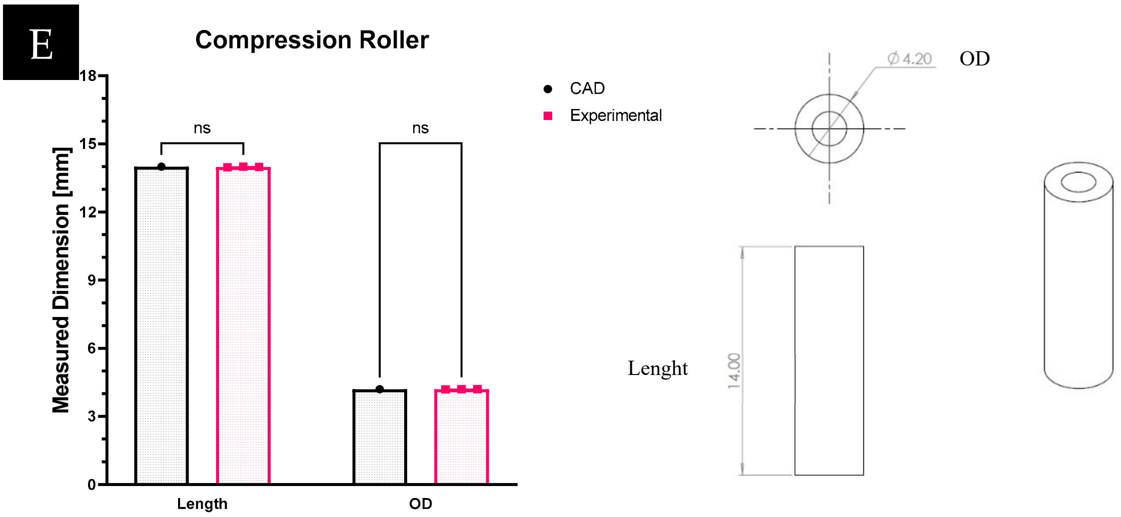

SI Figure 5. Characterizing accuracy of 3D printed components of the peristaltic pump assembly. Printing accuracy was evaluated on specific dimensions of each part. **(A)** Pump body. **(B)** Motor shaft adapter. **(C)** Bearing holder. **(D)** Tube holder. **(E)** Compression roller.

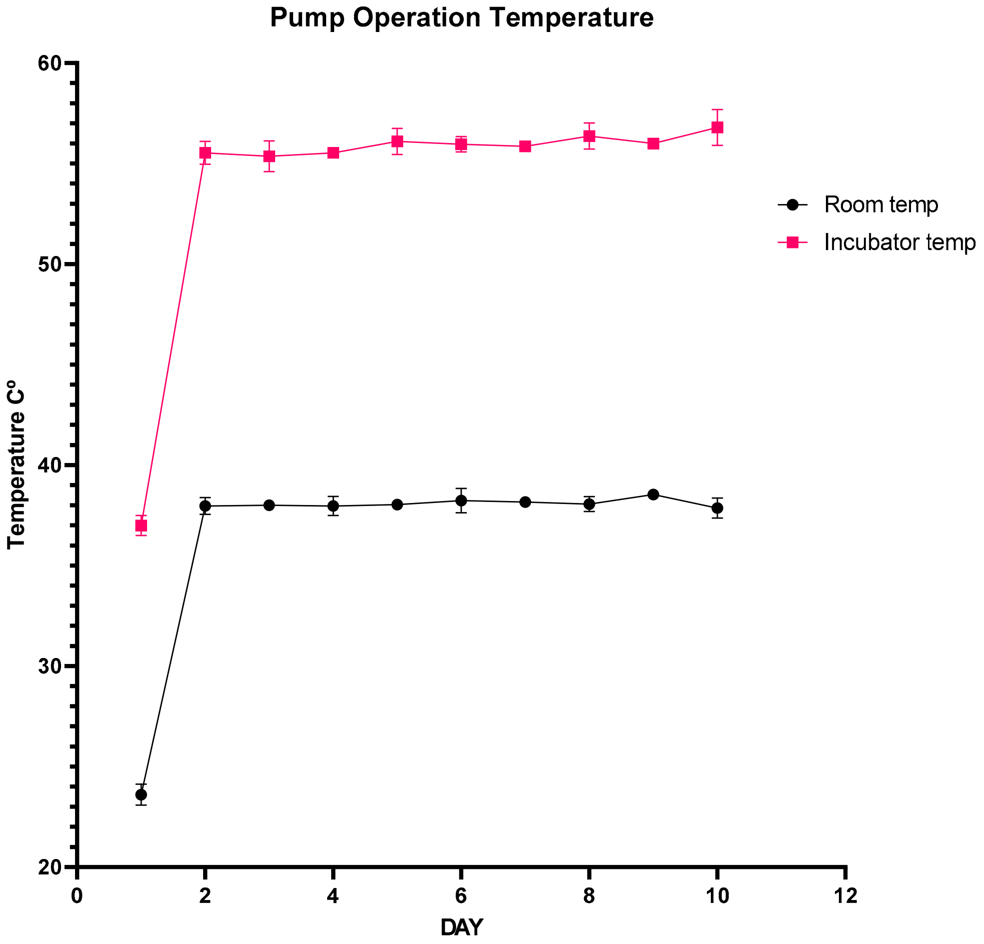

SI Figure 6. Pump Operation Temperature Measurements: temperature measurements obtained during a 10-day pump operation period at two different temperature conditions: room temperature and incubator temperature. The measurements were acquired three times a day on the contact face of the shaft side of the motor and the acrylic box.

Supplementary Methods

A- RPM constant program calculations

The delay is derived by finding an expression for the required pulse time needed for one motor step. If s is the number of steps needed for a full revolution of the motor, the delay required in seconds (noting that 1 revolution per second is 60 revolutions per minute) can be expressed as:

$$Delay = \frac{60}{RPM \times s} \tag{1}$$

Since two pulses (one high and one low) are needed to stop the motor, the delay in seconds can be expressed as:

$$Delay = \frac{60}{RPM \times s \times 2} = \frac{30}{RPM \times s} \tag{2}$$

If m is the micro stepping resolution or the number of micro steps each step is divided into, the expression becomes:

$$Delay = \frac{30}{RPM \times s \times m} \tag{3}$$

Since the motor requires 200 steps for 1 full revolution and the micro stepping resolution was 8 micro steps, the expression for the delay becomes:

$$Delay\ (s) = \frac{3}{RPM \times 160} \tag{4}$$

B- Pulsatile program calculations

A set of calculations were programmed for both the acceleration and deceleration component of the pulse. For these calculations, it is assumed that the desired output wave takes the form of a sawtooth wave, and thus the deceleration phase is significantly shorter than the increase (100:1). Additionally, it was assumed that there were fifty increments during ramping up and two increments during ramping down. The following set of equations (Equations 5 to 10) were used in the programmed algorithm loop to operate with the user interface.

$$Time\ for\ 1\ beat = \frac{60}{BPM} \tag{5}$$

$$AccelTime = \frac{T1\ Beat}{\#\ of\ increments} \tag{6}$$

$$AccelRate = \frac{Max\ Speed}{\#\ of\ increments} \tag{7}$$

$$Time\ for\ Decrease = \frac{T1\ Beat}{100} \tag{8}$$

$$AccelTimeDecrease = \frac{TDecrease}{\#\ of\ incrementsDecrease} \tag{9}$$

$$AccelRateDecrease = \frac{Max\ Speed}{\#\ of\ incrementsDecrease} \tag{10}$$
